# Cryotherapy for Rehabilitation After Total Knee Arthroplasty: A Comprehensive Systematic Review and Meta‐Analysis

**DOI:** 10.1111/os.14266

**Published:** 2024-10-14

**Authors:** Zhimin Liang, Zichuan Ding, Duan Wang, Yuchen Guo, Ling Zhu, Zeyu Luo, Lingli Li

**Affiliations:** ^1^ Department of Nursing West China Hospital/West China School of Nursing, Sichuan University Chengdu People's Republic of China; ^2^ Department of Orthopedics West China Hospital/West China School of Medicine, Sichuan University Chengdu People's Republic of China

**Keywords:** cryotherapy, systematic review and meta‐analysis, total knee arthroplasty

## Abstract

**Objective:**

Despite being well‐studied and widely utilized, the efficacy of cryotherapy after total knee arthroplasty (TKA) in enhancing early rehabilitation lacks consensus. The aim of this systematic review and meta‐analysis was to investigate (1) whether cryotherapy is able to promote the rehabilitation of patients undergoing TKA and (2) whether continuous cold flow device has superior results than cold pack in cryotherapy.

**Methods:**

A comprehensive trial searching was performed in the PubMed, Embase, Cochrane Library, and Google Scholar electronic databases in May, 2024. Randomized controlled trials (RCTs) comparing cryotherapy with no cryotherapy or comparing continuous cold flow device with cold pack after TKA were included. The primary outcome was visual analogue scale (VAS) of pain, and secondary outcomes included opioid consumption, blood loss (hemoglobin decrease and drainage), range of motion (ROM), swelling, length of stay (LOS), and adverse event.

**Results:**

A total of 31 RCTs were included in this meta‐analysis with 18 trials comparing cryotherapy with no cryotherapy and 13 trials comparing continuous cold flow device with cold pack. Pooled results showed cryotherapy group had significantly lower VAS scores than no cryotherapy group on postoperative day (POD) 1 (MD, −0.59 [95% CI, −1.14 to −0.04]; *p* = 0.04), POD 2 (MD, −0.84 [95% CI, −1.65 to −0.03]; *p* = 0.04), and POD 3 (MD, −0.86 [95% CI, −1.65 to −0.07]; *p* = 0.03). Cryotherapy group also showed reduced opioid consumption, reduced hemoglobin loss, decreased drainage, and improved ROM after TKA. Continuous cold flow device group had comparable VAS, opioid consumption, blood loss, ROM, knee swelling, and LOS with cold pack group.

**Conclusion:**

Cryotherapy can effectively alleviate postoperative pain, reduce blood loss, improve ROM, and thus promote the postoperative rehabilitation for TKA patients, but the continuous cold flow device did not show better efficacy than cold packs. These findings support the routine use of cryotherapy for the rapid rehabilitation of TKA patients, and the traditional cold pack is still recommended.

## Graphical abstract

Meta‐analysis showed that cryotherapy group had significantly lower visual analogue scale scores than no cryotherapy group on postoperative day (POD) 1 (MD, −0.59 [95% CI, −1.14 to −0.04]; *p* = 0.04), POD 2 (MD, −0.84 [95% CI, −1.65 to −0.03]; *p* = 0.04), and POD 3 (MD, −0.86 [95% CI, −1.65 to −0.07]; *p* = 0.03).

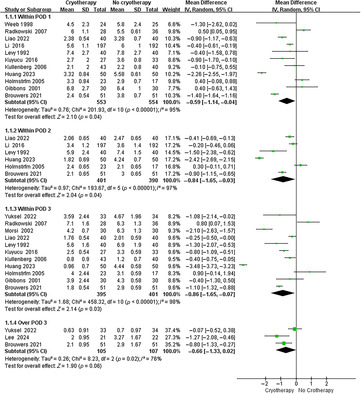

## 1 Introduction

Total knee arthroplasty (TKA) is widely acknowledged as the successful and efficacious treatment for end‐stage knee osteoarthritis, which offers excellent long‐term results including postoperative pain relief, deformity correction, and functional restoration for patients [1]. However, inflammatory response, blood loss and tissue damage in TKA procedure can lead to pain, swelling, and restricted range of motion (ROM) of the knee joint in early postoperative period, which significantly affect patients' recovery [2]. Given that early rehabilitation determines the long‐term functional outcomes after TKA, a series of perioperative managements has been promoted to enhance the rapid recovery after surgery [3, 4].

Cryotherapy, a widely employed non‐invasive therapeutic technique, involves the application of a cold substance to the skin surrounding surgical site. The types of commonly used cryotherapy devices are traditional cold pack and advanced continuous circulating cold water cuffs device. These current mainstream devices are widely used in clinical settings to maintain a constant temperature during cryotherapy. Despite being well‐studied and widely utilized, the efficacy of cryotherapy after TKA in enhancing early rehabilitation lacks consensus, with results from clinical trials remaining controversial [5, 6, 7]. Some studies reported that cryotherapy can alleviate pain by reducing nerve conduction velocity, and reduce blood loss and inflammatory response by stimulating vasoconstriction [8]. On the contrary, some studies found that transient intra‐articular temperature reduction does not effectively alleviate postoperative pain, bleeding and swelling after TKA [7]. It may even lead to delayed vasodilation and increased blood loss and swelling, while excessively low temperature or prolonged cooling is likely to induce severe pain and swelling in the surgical area [9].

By summarizing and analyzing the controversial results of published randomized controlled trials (RCTs), some previous systematic reviews have attempted to investigate the exact effectiveness of cryotherapy and compare various methods of cryotherapy after TKA [5, 7]. However, limited number of studies, lack of newly published high‐quality literatures, and absence of quantitative analysis have prevented these reviews from drawing a conclusion regarding the efficacy of cryotherapy after TKA. It is noteworthy that a considerable number of well‐conducted RCTs have been recently published [10, 11, 12, 13, 14, 15], which may help deepen our understanding of the application of cryotherapy and clarify its therapeutic effects after TKA. As a result, we performed this updated, comprehensive systematic review and meta‐analysis to investigate (1) whether cryotherapy is able to promote the rehabilitation of TKA patients and (2) whether continuous cold flow device has superior results than cold pack.

## 2 Methods

### 2.1 Study Design

This systematic review and meta‐analysis was conducted in accordance with the Cochrane Handbook for Systematic Reviews of Interventions and reported following the Preferred Reporting Items for Systematic Reviews and Meta‐Analyses (PRISMA) guidelines. The study protocol was registered with PROSPERO (CRD42024545008).

### 2.2 Trial Searching

A comprehensive trial searching was performed in the PubMed, Embase, Cochrane Library and Google Scholar electronic databases from the inception dates to May, 2024. No language restriction was applied. The full search strategy is shown in Table 1. Manual search of bibliographies from related reviewer and RCTs was also performed to identify published trials meeting inclusion criteria.

**TABLE 1:** Details of search strategy.

Database	Search strategy
Pubmed	#1 Arthroplasty, Replacement, Knee[MeSH] #2 (Knee arthroplasty[TIAB]) OR (Knee replacement[TIAB]) #3 #1 OR #2 #4 Cryotherapy[MeSH] #5 Cold Temperature[MeSH] #6 Cryo*[TIAB] OR Cold[TIAB] OR Cooling[TIAB] OR Icing[TIAB] OR Cold device[TIAB] OR Cold compress[TIAB] OR Cold Irrigation[TIAB] OR Cooling pad[TIAB] OR Computer assisted cold therapy[TIAB] OR Ice bag[TIAB] OR Ice pack[TIAB] OR cold gases[TIAB] OR Ice pad[TIAB] #7 (#4 OR #5 OR #6) #8 (#3 AND #7)
Embase	#1 ‘Knee arthroplasty’/exp #2 ‘Knee arthroplasty’:ab,ti OR ‘Knee replacement’:ab,ti #3 #1 OR #2 #4 ‘Cryotherapy’/exp #5 ‘Cold’/exp #6 ‘Cryo*’:ab,ti OR ‘Cold’:ab,ti OR ‘Cooling’:ab,ti OR ‘Icing’:ab,ti OR ‘Cold device’:ab,ti OR ‘Cold compress’:ab,ti OR ‘Cold Irrigation’:ab,ti OR ‘Cooling pad’:ab,ti OR ‘Computer assisted cold therapy’:ab,ti OR ‘Ice bag’:ab,ti OR ‘Ice pack’:ab,ti OR ‘cold gases’:ab,ti OR ‘Ice pad’:ab,ti #7 (#4 OR #5 OR #6) #8 (#3 AND #7)
Cochrane Library	#1 MeSH descriptor: [Arthroplasty, Replacement, Knee] explode all trees #2 (Knee arthroplasty):ti,ab,kw OR (Knee replacement):ti,ab,kw #3 (#1 OR #2) #4 MeSH descriptor: [Cryotherapy] explode all trees #5 MeSH descriptor: [Cold Temperature] explode all trees #6 (Cryo*):ti,ab,kw OR (Cold):ti,ab,kw OR (Cooling):ti,ab,kw OR (Cold device):ti,ab,kw OR (Cold compress):ti,ab,kw OR (Cold Irrigation):ti,ab,kw OR (Cooling pad):ti,ab,kw OR (Computer assisted cold therapy):ti,ab,kw OR (Ice bag):ti,ab,kw OR (Ice pack):ti,ab,kw OR (Cold gases):ti,ab,kw OR (Ice pad):ti,ab,kw #7 (#4 OR #5 OR #6) #8 (#3 AND #7)
Google Scholar	(Cryo* OR Cold OR ice OR Cooling OR Cold compress OR Cooling pad OR Ice bag OR Ice pack OR cold gases OR Ice pad) AND (Knee arthroplasty OR Knee replacement)

### 2.3 Trial Selection

Following inclusion criteria were used for trial selection: (1) RCTs; (2) trials enrolling patients underwent primary TKA; (3) trials comparing cryotherapy with no cryotherapy or comparing continuous cold flow device with cold pack; (4) interventions besides cryotherapy were the same in both groups in the trials; and (5) trials reporting rehabilitation data after TKA, such as pain, opioid consumption, swelling, ROM, blood loss and hospital length of stay (LOS).

Two independent reviewers assessed the titles and abstracts for the initial screening of trials. Subsequently, the full texts of trials selected from the initial screening were evaluated. Trials meeting the inclusion criteria were ultimately included. In the trial selection process, if a decision could not be reached, the opinion of a third reviewer was sought.

### 2.4 Assessment of Risk of Bias

The risk of bias of RCTs was assessed using Revised Cochrane Risk of Bias Tool for randomized trials (RoB 2) based on the following five domains: bias arising from the randomization process; bias due to deviations from intended interventions; bias due to missing outcome data; bias in measurement of the outcome; and bias in selection of the reported result. Each domain consists of several sub‐items, and a proposed judgment about the risk of bias arising from each domain is based on answers to the sub‐items. Judgments can indicate either a low or high risk of bias, or express as some concerns. Two reviewers independently evaluated the risk of bias in the included trials, with any discrepancies resolved by a third reviewer.

### 2.5 Data Extraction

Two authors independently extracted the following information from the included trials: lead author, country, sample size, age of participates, gender of participates, type of intervention, intervention protocol, outcomes and study results. Study outcomes related to the postoperative rehabilitation were extracted and categorized into several aspects: pain (including VAS [visual analogue scale] and opioid consumption), blood loss (including hemoglobin decrease and drainage), ROM, swelling, hospital LOS and adverse event.

VAS was defined as the primary outcome of this study, with the scale of 0 indicating “no pain” and the scale of 10 indicating “pain too intense to be tolerated.” The opioid consumption refers to the amount used within 72 h postoperatively, with the unit standardizing to mg/kg. The unit of hemoglobin decrease was standardized to g/L. Knee swelling refers to the difference in knee circumference postoperatively compared to preoperatively, measured in centimeters. All reported adverse events were recorded.

### 2.6 Statistical Analysis

Meta‐analyses in this study were performed using Review Manager (version 5.4.1, the Cochrane Collaboration, the United Kingdom). For continuous outcomes, mean difference (MD) and 95% confidence intervals (CI) were calculated using the Inverse Variance (IV) method. For discrete outcomes, odd ratio (OR) and 95% CI were calculated using the Mantel–Haenszel (M‐H) method. Heterogeneity among studies was assessed using the *I* ^2^ statistic, with values exceeding 50% indicating substantial heterogeneity. Studies with significant heterogeneity were pooled up using random effect model, otherwise a fixed effect model was used. When standard deviation (SD) was not reported in the original study, an imputed SD was calculated in accordance with recommendations from the Cochrane Handbook. In cases where measurement scales varied, units were standardized to the metric system. To investigate whether the clinical impact of cryotherapy varies over time, the outcomes underwent multiple analyses based on different follow‐up times. Data from the nearest available time point were utilized for analysis. To evaluate whether the pain relief effect of cryotherapy was associated with the type of cryotherapy device, we performed subgroup analysis based on the cryotherapy device (continuous cold flow device and cold pack vs. no cryotherapy). Statistical significance was set as *p* < 0.05 in this study.

## 3 Results

### 3.1 Trials Selection and Characteristics

**FIGURE 1:**
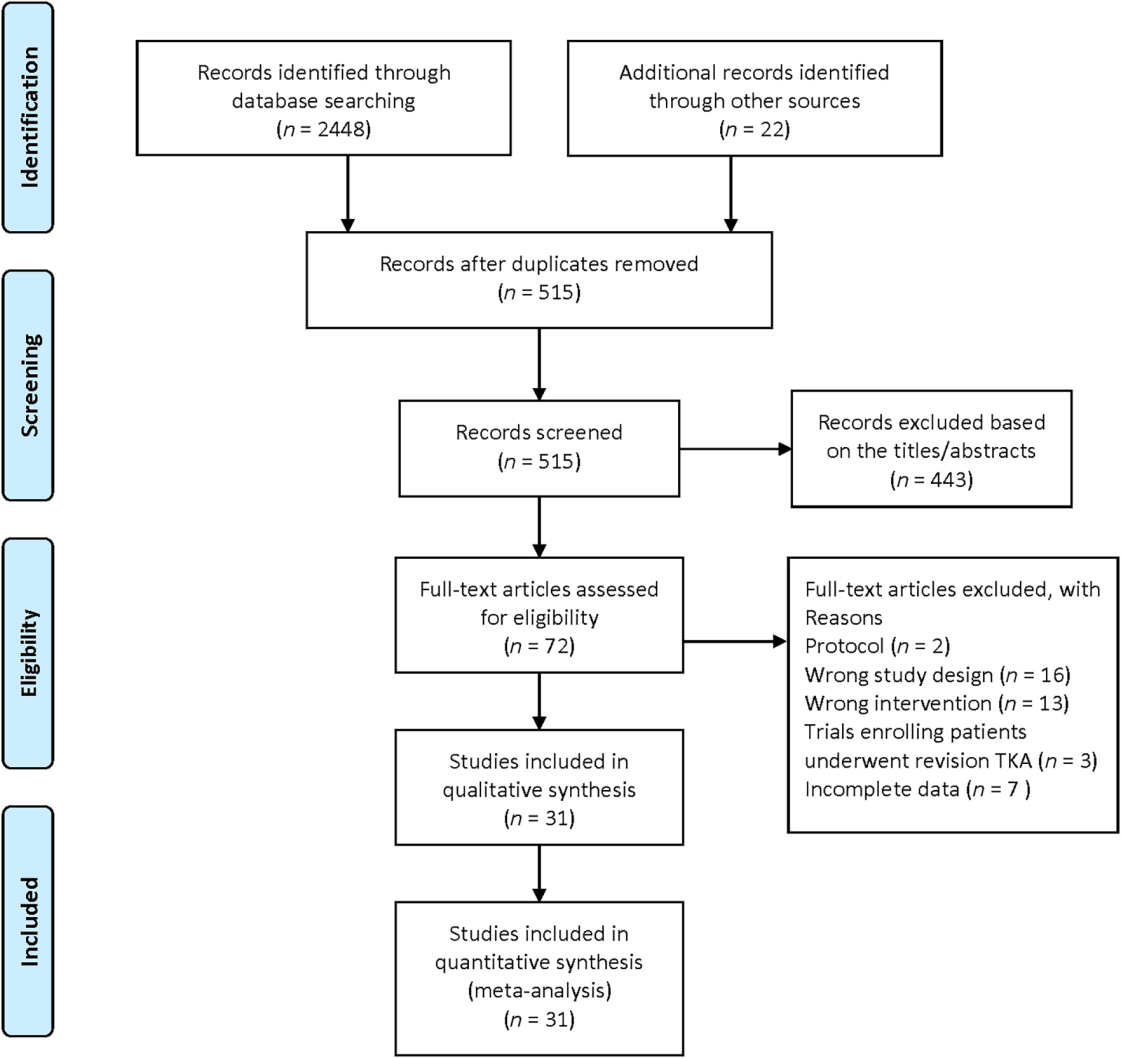
Flow diagram of included studies.

The initial literature search from electronic databases identified 2448 trials and the manual search yielded 22 additional trials. After removed duplicates, a total of 515 trials remained. After excluded 443 trials based on the titles/abstracts screening, the full‐text of 72 trials were assessed for eligibility. Then, we excluded 41 trials for the reasons listed in Figure 1. Finally, 31 RCTs that met the inclusion criteria were included in this meta‐analysis.

A total of 18 trials compared cryotherapy with no cryotherapy, and their characteristics are summarized in Table 2 [12, 13, 14, 16, 17, 18, 19, 20, 21, 22, 23, 24, 25, 26, 27, 28, 29, 30]. The devices used in cryotherapy groups included cold pack, circulating cold water device, computerized control of continuous cold therapy device, ice thermal pads and cryogenic liquid carbon dioxide gas. The number of trials utilizing continuous cryotherapy was 15, while others utilized non‐continuous cryotherapy. Trials employing cryotherapy devices with pressure capabilities totaled 7, whereas others used devices without pressure capability. Trials utilizing cryotherapy devices with temperature control amounted to 6. Regarding the timing and frequency of cryotherapy, except for Kuyucu et al., which initiated cryotherapy 2 h before surgery [22], and Li et al., which initiated cryotherapy intraoperatively [24], all other included studies initiated cryotherapy on the day of surgery. Frequency of cryotherapy ranged from once daily to thrice daily or were continuous uninterrupted, with durations varying from 2 days to 3 weeks.

**TABLE 2:** Characteristics of included trials comparing cryotherapy with no cryotherapy.

Source	County	Participates (intervention group/control group)			Intervention group (cryotherapy)			Control group (no cryotherapy)	Outcome	Favor
		Sample size	Age, mean (SD), year	Women, %	Device	Start time	Frequency			
Brouwers et al., 2022	Netherlands	51/51	69.6 (9.1)/69.2 (6.8)	29 (56.9)/29 (56.9)	Computer‐assisted cryotherapy (ZAMAR, Vrsar, Croatia)	Applied immediately after surgery	Applied three times a day at set temperature for 7 days after surgery	Received the usual rapid recovery care and rehabilitation program	Pain (NRS) Opioids consumption Swelling: circumference of knee Satisfaction: numerical rating scale for satisfaction Complication ROM, TUG, KOOS, WORQ	Cryotherapy
Gibbons et al., 2001	UK	30/30	71/71	19 (63.3)/16 (53.3)	Cryo/Cuff (Aircast, UK)	Return to the ward after surgery	The device was used for a minimum of 6 h per day throughout the hospital stay	Received a modified Robert Jones bandage within 48 h after surgery	Blood loss: total drain output ROM Pain (VAS) Morphine consumption LOS; Adverse events	No cryotherapy
Holmstrom et al., 2005	Sweden	23/17	68 (7)/72 (6.5)	9 (39)/6 (35)	Cryo/Cuff	Applied immediately after skin closure and before release of the tourniquet	The treatment continued without interruption for 48 h. The ice water changed every sixth hours	Received only traditional analgesics within 48 h after surgery	Pain (VAS) Morphine consumption ROM Swelling Blood loos: total drain output Adverse events	Cryotherapy
Huang et al., 2023	China	50/50	65.8 (7)/64.6 (6)	35 (70)/34 (68)	Continuous cold flow device	Applied from 4 h after surgery	Cryotherapy performed 90 min per 120 min until 7 days after surgery. Received an intra‐articular injection of normal saline (10 mL) by drainage at 4 h after operation and then clamped	Received an intra‐articular injection of normal saline (10 mL) by drainage at 4 h after operation and then clamped	Pain (VAS) Blood loss: intraoperative blood loss, total drain output, Hb decrease LOS; Complications Analgesia consumption Function score (HSS)	Cryotherapy
Ivey et al., 1994	USA	28/30	64.5 (8.1)/66.9 (11.6)	16 (57.1)/22 (73.3)	Hot Ice thermal pads (Pro‐Action Medical, Oak Ridge, TX)	Applied immediately after surgery	Applied throughout 72 h after surgery with The temperature of 50 °F	Received same device with the temperature of 70 °F	Morphine consumption	No cryotherapy
Kang et al., 2014	Korea	16/15	67.6 (3.5)/66.6 (3.9)	\	Cryotherapy Crais (Century, Korea) &Ultrasound therapy (Care Star GM‐002)	Applied immediately after surgery	Cryotherapy lasted for 5 min, low‐intensity pulsed ultrasound therapy lasted for 1 min, the above two treatments were administered once a day, 5 times a week for 3 weeks	Received low‐intensity pulsed ultrasound therapy once a day, 5 times a week for 3 weeks	K‐WOMAC ROM CRP	Cryotherapy
Kullenberg et al., 2006	Sweden	43/40	68 (6)/69 (7)	25 (58)/24 (60)	Cryo/cuff (Aircast, Summit, NJ)	Applied immediately after skin closure	The device was rechilling every 60 min by the nursing staff for 3 days (mean 60 h) until the cold compression dressing was removed	Received normal routine care: epidural analgesia (EDA) with ropivacaine until POD3, anti‐inflammatory drugs and opioids	Pain (VAS) ROM Opioids consumption Blood loos: hemoglobin LOS; Adverse events	Cryotherapy
Kuyucu et al., 2015	Turkey	27/33	67.2 (5.25)/68.4 (6.25)	\	Cryo/Cuff	Applied 2 h before surgery	Repeated in postoperative 6th hour, and applied 2 h every day, during postoperative 4 days	Received routine analgesics and care	Pain (VAS) Blood loss: hemoglobin values, total drain output KSS score	Cryotherapy
Lee et al., 2024	Korea	21/22	72.4 (5.8)/71 (7.2)	18 (85.7)/18 (81.8)	Cryogenic liquid carbon dioxide gas at −78°C	Applied from POD3	Applied six times a week for 2 weeks, and once each before and after exercise for 3 min	Completed daily 30 min exercise sessions, once a day, for a total of 12 sessions over a 2‐week period	Pain (VAS) ROM Swelling: knee Circumference Function score (10 MWT)	Cryotherapy
Levy AS et al., 1993	USA	40/40	74/73	33 (82)/32 (80)	Aircast Cryo/Cuff	Applied immediately after surgery	Rechilling was performed every 90 min by the nursing staff for 3 days until the dressing Was removed	The incision covered with gauze pads, four layers of webril, and an ace from toe to thigh before tourniquet deflation for 3 days	Blood loos: total drain output , hemoglobin decrease Pain (VAS) Morphine consumption ROM Swelling: knee Circumference	Cryotherapy
Li et al., 2016	China	197/192	60.7 (6.5)/61.8 (5.8)	169 (86)/167 (87)	Cold water irrigation	Continuous irrigation of either 4000 mL cold saline with 0.5% epinephrine interoperate. After closing the joint capsule, 50 mL of cold irrigation solution with 0.5% epinephrine was administered through a drainage tube placed in the knee joint cavity		Continuous irrigation of 4000 mL normal saline at normal temperature, after closing the joint capsule, 50 mL of normal temperature saline was administered through a drainage tube placed in the knee joint cavity	Pain: VAS Swelling: Knee circumference Blood loss: total drain output, decreased hemoglobin Morphine consumption Sleep quality (VAS) Satisfaction rate (VAS) Complications	Cryotherapy
Liao et al., 2022	China	40/40	64.8 (5.4)/65.3 (5.4)	24 (60)/22 (55)	Ice packs and ERAS	Applied from POD 0	Null Information	Received routine care. No special heat preservation method was adopted during the surgery	Pain (VAS) ROM Function score (HSS) Self‐care ability (Barthel index) Quality of life (SF‐36) Rehabilitation time Cost The time to out‐of‐bed activities Complications	Cryotherapy
Morsi et al., 2002	Egypt	30/30	\	\	Continuous‐flow cooling device	Started after skin closure and before tourniquet	Applied continuously for 6 days postoperatively except for short periods for ambulatory and knee mobilization exercises	Received routine care but no cooling device	Pain (VAS) ROM Blood loss: total drain output Analgesic consumption Complications	Cryotherapy
Radkowski et al., 2007	USA	28/36	63.7 (10.4)/66.9 (10.4)	13 (46)/23 (64)	Thermo‐Tek Solid State Recirculating Chiller (Thermo‐Tek Inc., Carrolton, TX)	Applied from POD 1	Applied from POD 1 to discharge at 45 °F	Applied from POD1 to discharge at 75 °F	Pain: VAS Narcotic consumption Blood loss: total drain output Complications	No cryotherapy
Thijs et al., 2018	The Netherlands	30/30	65.5 (6.2)/64.7 (6.8)	13 (43.3)/15 (50.0)	Computer‐assisted cryotherapy (Zamar Therapy Cube, Vrsar, Croatia)	Applied immediately after surgery	Applied during the first 7 days after TKA with a fixed protocol at 10°C–12°C	Received the same cooling protocol but with another temperature setting of 21°C	Pain (NRS) Opioids consumption Swelling: knee Circumference ROM WOMAC EuroQoL‐5D	Cryotherapy
Walker et al., 1991	USA	15/15	75/70	\	Continuous cooling pad (elastic mesh American Hospital Supply Corp) & CPM	Applied begun in recovery room	Continuous cooling pad applied at least 3 days after surgery with the temperature of 50–55 °F. CPM was continued throughout hospitalization	CPM was continued throughout hospitalization	LOS Blood loss: total drain output ROM Analgesia consumption	Cryotherapy
Webb et al., 1998	UK	24/24	69/70.9	\	Cryo/Cuff (aircast)	Applied immediately after surgery	Null Information	Received the wool and crepe dressing immediately after surgery	Blood loss: total drain output Pain (VAS) Analgesia consumption Swelling: knee Circumference ROM	Cryotherapy
Yuksel et al., 2022	Turkey	33/34	66.8 (9.80)/65.4 (8.94)	23 (70)/23 (68)	Cold packs	Applied immediately after surgery	Applied 12–15 min every 2 h throughout inpatient rehabilitation period (mean 6.08 days)	Received the same standard postoperative rehabilitation protocol	Pain (NRS) Swelling: frustum formula Muscle strength: Lafayette Manual Muscle Testing System. TUG; 10MWT; ILAS; HSS Knee score, ROM Quality of life (SF‐12)	Cryotherapy

Abbreviations: 10MWT, 10‐m walk test; CRP, C‐reactive protein; ERAS, enhanced recovery after surgery; *EuroQol*‐5D, EuroQol five dimensions questionnaire; HSS Knee score, hospital for special surgery knee score; ILAS, Iowa level of assistance scale; KOOS, Knee Injury and Osteoarthritis Outcome Score; KSS, Knee Society Scoring System; K‐WOMAC, Korean Western Ontario and McMaster Universities Arthritis Index; LOS, length of hospital; NRS, Numeric Rating Scale; POD, postoperative day; ROM, range of motion; SF‐12, short Form 12 health survey; SF‐36, the MOS item short from health survey; TUG, timed‐up and go; VAS, visual analogue scale; WORQ, Work, Osteoarthritis and Joint‐Replacement Questionnaire.

A total of 13 trials compared continuous cold flow device with cold pack and their characteristics are summarized in Table 3 [10, 15, 31, 32, 33, 34, 35, 36, 37, 38, 39, 40, 41]. Trials employing continuous cryotherapy devices with pressure capabilities totaled 5, whereas those without pressure capability amounted to 8. Trials utilizing continuous cryotherapy devices with temperature control amounted to 10. In terms of the initiation timing of cryotherapy, except for Demoulin et al., which initiated cryotherapy on POD 2 [33], all others initiated cryotherapy on the day of surgery. Frequency of cryotherapy ranged from 2 to 6 times daily or were continuous uninterrupted, with durations mostly within a span of 2 weeks.

**TABLE 3:** Characteristics of included trials comparing continuous cold flow device with cold pack.

Source	County	Intervention group/control group			Intervention group (continuous cryotherapy)			Control group (cold pack)	Outcome	Favor
		Sample size	Age, mean (SD), year	Women, %	Device	Start time	Frequency			
Bech et al., 2015	Canada	37/34	70.4 (1.8)/71.5 (1.8)	20 (54)/14 (42.1)	Icing device (Don Joy Iceman, DJO Canada, Mississauga, ON)	Applied immediately after surgery	Remained in place for 48 h, except for brief periods	Received ice bags at a frequency requested by the patient (usual care) for 48 h	Pain (NRS) ROM; WOMAC; Vomiting Opioids consumption Blood loss: hemoglobin decrease Compliance and satisfaction Adverse events; LOS	Cold pack
Borgers et al., 2020	Belgium	26/26	69.12 (9.0)/69.15 (9.7)	19 (73) 17 (65)	Continuous cold flow device (Vascutherm 4 (Thermo Tek Inc.))	Applied when the patient was discharged from recovery room	A maximum of 6 cooling cycles was applied per day at 10°C	Cold pack Start applied when the patient is discharged from the recovery room. Each cold treatment lasted 20–30 min, 4 times a day	Pain (NRS) Swelling: knee circumference ROM: POD1‐15 Analgesic consumption Satisfaction: 6‐point verbal scale Number and times of cooling	Continuous cryotherapy
Demoulin et al., 2012	Belgium	22/22	68.2 (10.3)/71.1 (7.51)	13 (59)/13 (59)	Water circulating device (Aircast1 Cryocuff1, Inc., Summit, New Jersey) and the Auto Chill1 System (Aircast1, Inc., Summit, New Jersey)	Applied from POD2 to last hospitalization day	Applied to the knee for 20 min, five times a day	Applied for 20 min, five times a day from POD2 to last hospitalization day.	Pain (VAS) Swelling: knee circumference ROM Cutaneous temperature	Cold pack
Desteli et al, 2015	Turkey	42/45	65.1 (4.1)/65.4 (7.0)	20 (47.6)/23 (51.1)	Computerized control of continuous cold therapy (C‐Tx)	Applied from the POD 0	Applied to the operated knees preoperatively for 90 min until they were taken to the operation hall and postoperatively for 6 h on surgery day starting just after the operation, and for 2 h for subsequent 2 days	Did not take any cold therapy before the operation. Received ice packages 8 times for 15 min with 45‐min intervals for the operation day and postoperative 2nd day	Pain (NRS) Blood loss: hemoglobin, total Drain output	Continuous cryotherapy
Healy et al., 1993	USA	50/55	\	\	Cold compressive dressing (Cryo/Cuff)	Applied after operation	Null Information	Received ACE wrap and ice packs after operation	Swelling: knee circumference Narcotic consumption Blood loss: total Drain output ROM, Adverse events	Cold pack
Karaduman et al., 2019	Turkey	30/29	62.7 (8.8)/65.5 (8.6)	26 (86.7)/26 (89.7)	Continuous cold flow device (Waegener, Beerse, Belgium)	Applied immediately after surgery until POD3	On the POD 0 it was Applied for the first 6 h. On the POD1, it was applied at 2 h intervals. On the POD2 and POD3 it was applied every 6 h for 2 h	A cold pack (gel ice) was applied as standard treatment for 20 min every 2 h for 3 days postoperatively	Pain (NRS) Blood loss: hemoglobin decrease Swelling: knee circumference ROM knee temperature	Continuous cryotherapy
Marinova et al., 2023	Australia	36/36	64.7 (10.1)/68.9 (8.8)	23 (63.9)/17 (47.2)	Game Ready (GRPro 2.1 cryo compression system)	Applied on POD0 upon their return to the ward continued 2 weeks	Applied 20 min at a time, 6 times a day, at least 1‐h intervals, for 2 weeks	Applied regular ice bag along with tubigrip static compression applied under the ice. use 20 min at a time, 6 times a day, at least 1‐h intervals for 2 weeks	Pain (VAS) ROM Opioids consumption LOS; KOOS score	Cold pack
Quesnot et al., 2024	France	20/20	76.2 (6.2)/76.7 (8.8)	2 (10)/7 (35)	Game Ready (Cool systems, Inc. Concord, CA, USA).	Applied from rehabilitation Day 1 to day 21	Applied for 30 min, three times per day, 5 days per week throughout the study period	Ice wraps and ice wraps were applied for 30 min, three times per day, 5 days per week throughout the study period	ROM Swelling: knee circumference Joint effusion Pain (VAS) Walking distance (6MWT) patient's independence (KOOS)	Continuous cryotherapy
Ruffilli et al., 2017	Italy	24/ 26	\	18 (70.6)/9 (35)	Continuous cold flow device (Hilotherm GmbH, Germany)	Applied immediately after surgery until discharge	Ice cold packs were applied over the bandage immediately after surgery and changed every 30 min. Then the Hilotherm device were applied over the skin on POD1 at 12°C	Cold pack applied directly in the operating room, and changed every 30 min until discharge	Pain (NRS) Swelling: knee circumference Analgesic consumption ROM; LOS Blood loss: drain output, transfusion requirement Complications	Cold pack
Sadoghi et al., 2017	Austria	46/51	70.35/71.67	29 (63)/32 (62)	Continuous cold flow (cTreatment system)	Applied from the POD 0	Applied 1 h on the POD0, then applied 6 h in total in the post anesthesia care unit. During the in‐patient setting until the sixth POD, the device was applied each day for 4 h in total, 2 h in the morning and 2 h in the afternoon	Did not received cryotherapy before the surgical intervention then received cold pack 6 days after the surgical intervention. Applied three times per day for 20 min each throughout the whole trial	Pain (NRS) ROM Swelling: knee circumference Hydromorphone consumption Adverse events LOS	Continuous cryotherapy
Schinsky et al., 2016	USA	48/49	65.33/64.65	25 (53)/32 (60)	Circulating cold water device (Polar Care Glacier; Breg, Inc.)	Start applied on the recovery room	The cryotherapy device was applied from POD1 to POD11, and treatment plan was divided into three stages, each of which performs a different cryotherapy regimen	Received cold pack from recovery room and replaced every 3–4 h. The use time is the same as that of the experimental group	Pain (VAS); ROM Morphine consumption HGB and HCT Swelling: knee circumference Blood loss: total drain output Compliance and Satisfaction Adverse events LOS	Continuous cryotherapy
Su et al., 2012	USA	103/84	\	\	Cryopneumatic device (The GameReady device (Cool Systems, Inc.))	Applied within 3 h of surgery	Applied for 2 h on, and 1 h off for a minimum of four cycles per day. After discharge, the application time was 1 h on, and 30 min off until 2 weeks after surgery	Applied ice bag with static compression. The use time is the same as that of the experimental group	Pain (VAS) Swelling: knee circumference Narcotic consumption ROM 6MWT TUG Satisfaction Adverse events	Continuous cryotherapy
Thienpont et al., 2014	Belgium	50/50	67.5 (10.5)/68.5 (10)	35 (70)/40 (80)	Advanced cryotherapy (cTreatment)	Applied in the recovery room and continued in their room	Patients received 4 h of continuous cooling at 11°C immediately after surgery, On POD1 the cTreatment Applied 2 h in the morning and afternoon. During the evening and night, patients were allowed to use the cTreatment when they considered it's necessary	Applied 15 min on arrival to the recovery room and again on arrival to the ward. Repeated 2 h and 4 h after surgery. The following days received the same cold pack cryotherapy 15 min after their physiotherapy session	Pain (VAS) Analgesics consumption ROM Swelling: knee circumference Blood loss: hemoglobin decrease Adverse events LOS	Cold pack

Abbreviations: 6MWT, 6‐m walk test; HCT, hematocrit; HGB, hemoglobin; KOOS, Knee Injury and Osteoarthritis Outcome Score; LOS, length of hospital; NRS, Numeric Rating Scale; POD, postoperative day; ROM, range of motion; TUG, timed up and go; VAS, visual analogue scale; WOMAC, Western Ontario and McMaster Universities Arthritis Index.

**FIGURE 2:**
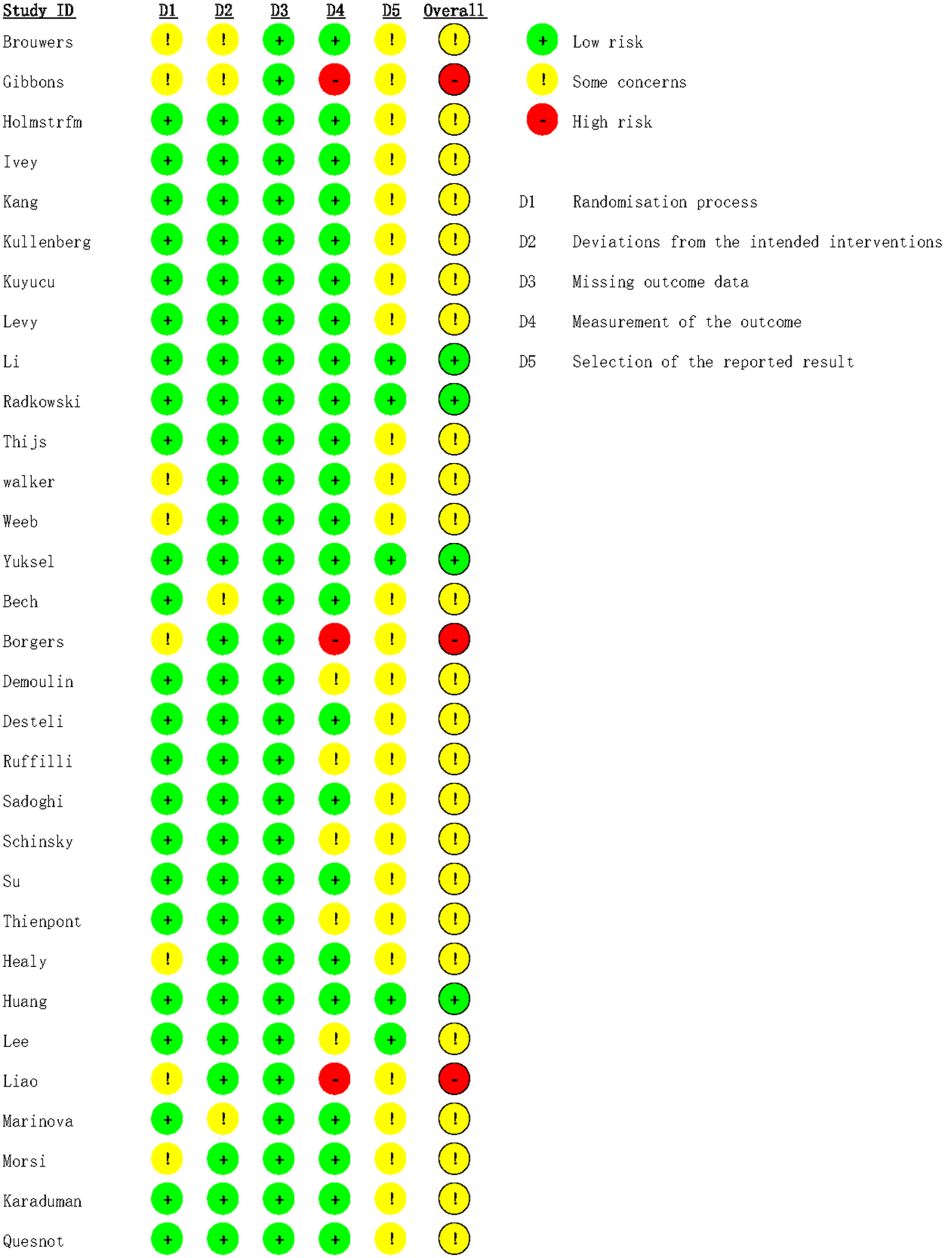
Risk of bias for individual trials.

**FIGURE 3:**
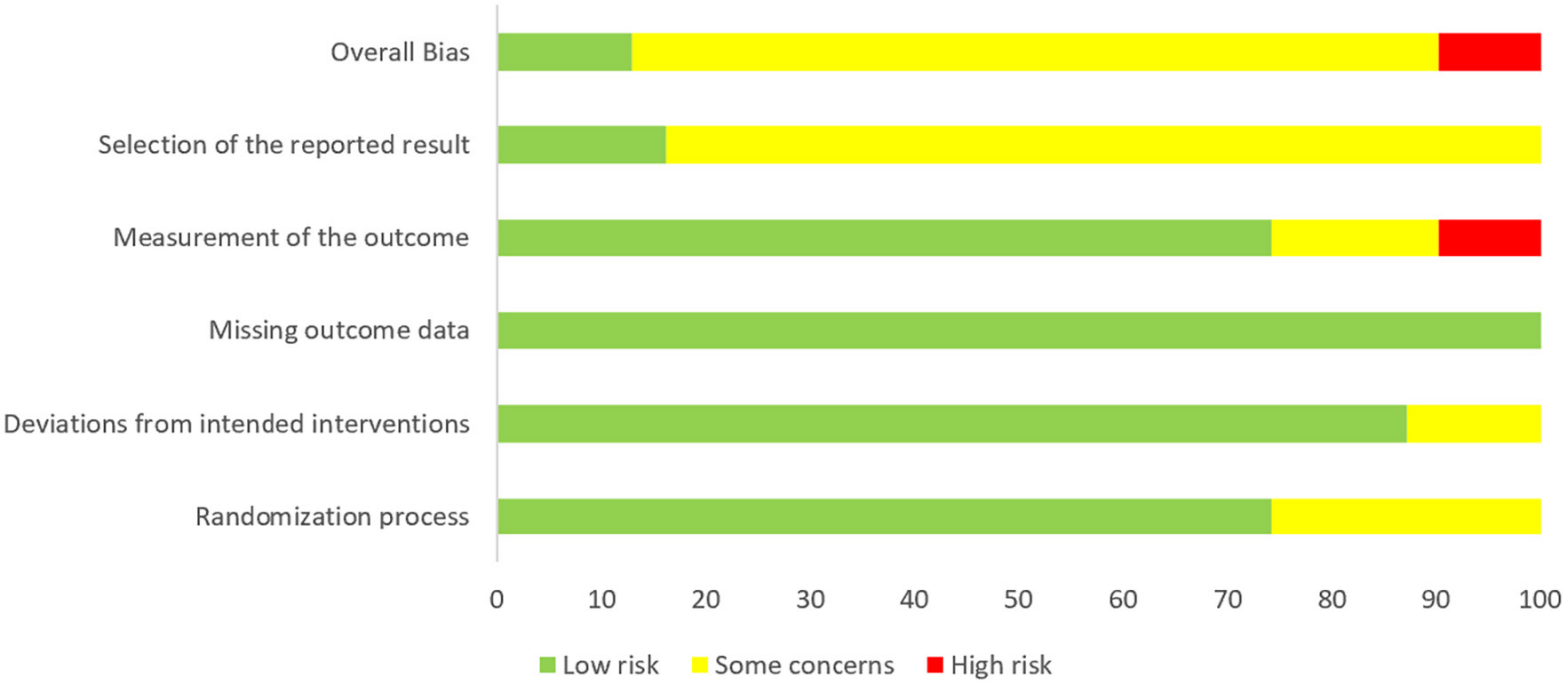
Summary of risk of bias of included trials.

Details of risk of bias of included trials measured by RoB 2 were shown in Figures 2 and 3. The results revealed that three trials were assessed as high risk, four trials as low risk, and the remaining trials were evaluated as some concerns. The risk of bias stemmed from lacking clear allocation concealment during the randomization process, executing outcome measurements without blinding, and the inability to determine the risk of selection of the reported result due to the absence of registration information.

### 3.2 Cryotherapy vs. No Cryotherapy

#### 3.2.1 Pain

**FIGURE 4:**
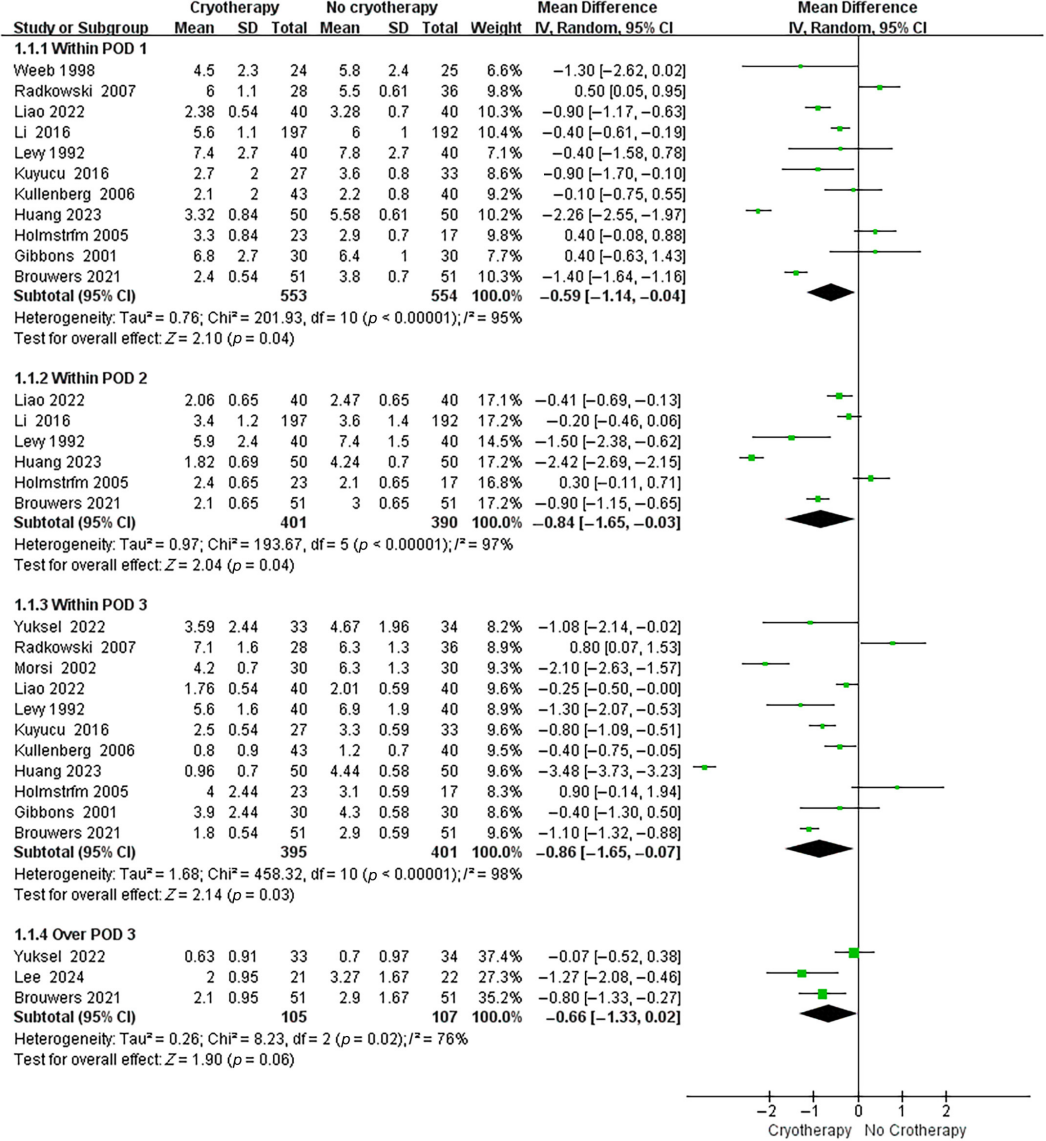
Forest plot for pain score (cryotherapy vs. no cryotherapy).

**FIGURE 5:**
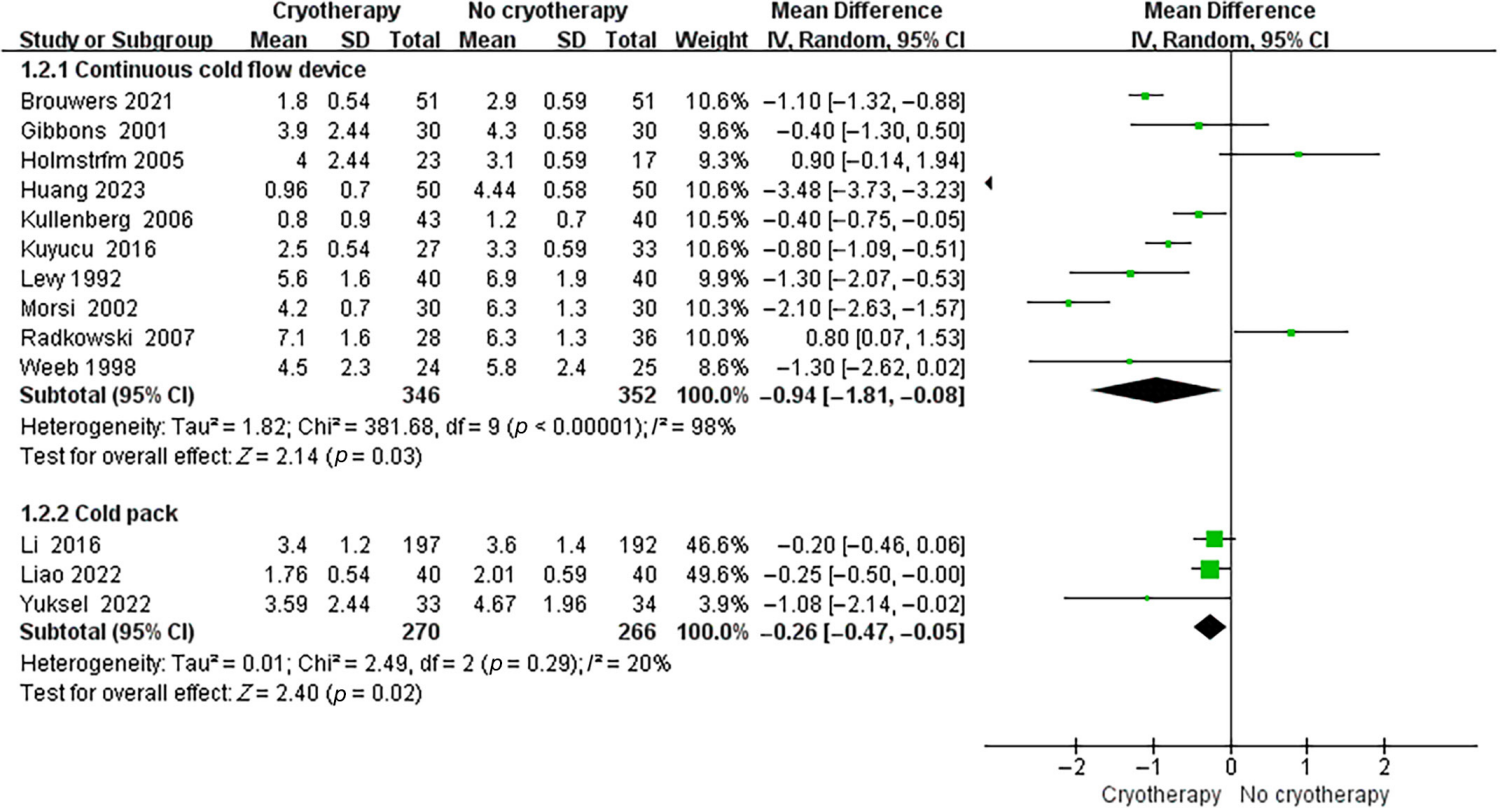
Subgroup analysis forest plot for pain score (cryotherapy vs. no cryotherapy).

Regarding the use of cryotherapy to alleviate postoperative pain following TKA, 11 trials assessed the visual analog scale (VAS) on postoperative day (POD) 1, 6 trials assessed the VAS on POD 2, 11 trials assessed the VAS on POD 3, and 3 trials assessed the VAS over POD 3. As shown in Figure 4, cryotherapy group had significantly lower VAS scores than no cryotherapy group on POD 1 (MD, −0.59 [95% CI, −1.14 to −0.04]; *p* = 0.04), POD 2 (MD, −0.84 [95% CI, −1.65 to −0.03]; *p* = 0.04), and POD 3 (MD, −0.86 [95% CI, −1.65 to −0.07]; *p* = 0.03). No significant difference was observed on VAS between groups over POD 3 (MD, −0.66 [95% CI, −1.33 to 0.02]; *p* = 0.06). In subgroup analysis, continuous cold flow device and cold pack both showed significant pain relief effect when compared to no cryotherapy (Continuous cold flow device vs. no cryotherapy: MD, −0.94 [95% CI, −1.81 to −0.08]; *p* = 0.03. Cold pack vs. no cryotherapy: MD, −0.26 [95% CI, −0.47 to −0.05]; *p* = 0.02) (Figure 5).

A total of 5 studies compared the postoperative opioid consumption between two groups, which is a crucial indicator reflecting postoperative pain management after TKA. The result demonstrated that cryotherapy group had significantly reduced opioid consumption when compared to no cryotherapy group (MD, −0.09 [95% CI, −0.15 to −0.02]; *p* = 0.009) (Figure S1).

#### 3.2.2 Blood Loss

Five trials involving 692 patients compared the postoperative hemoglobin decrease between cryotherapy and no cryotherapy groups. The results showed that cryotherapy group had significantly less hemoglobin decrease than no cryotherapy group (MD, −0.74 [95% CI, −1.36 to −0.13]; *p* = 0.02) (Figure S2).

For the association between cryotherapy and drainage after TKA, 11 trials with 485 patients receiving cryotherapy and 489 patients receiving no cryotherapy were pooled. As demonstrated in Figure S3, cryotherapy significantly reduced the drainage when compared to no cryotherapy (MD, −126.99 [95% CI, −190.52 to −63.47]; *p* < 0.00001).

#### 3.2.3 ROM

To investigate whether cryotherapy could improve the postoperative ROM after TKA and whether the effect varied over time, ROM within POD 7 and over POD 7 between cryotherapy and no cryotherapy groups were investigated. Cryotherapy group had a higher ROM than no cryotherapy group both within POD 7 (MD, 6.26 [95% CI, 3.88 to 8.64]; *p* < 0.00001) and over POD 7 (MD, 5.84 [95% CI, 3.51 to 8.17]; *p* < 0.00001) (Figure S4).

#### 3.2.4 Swelling

The association between cryotherapy and postoperative knee swelling was evaluated. Cryotherapy and no cryotherapy groups had similar knee swelling parameters either within POD 7 (MD, −0.56 [95% CI, −1.33 to 0.21]; *p* = 0.16) or over POD 7 (MD, 0.55 [95% CI, −0.69 to 1.79]; *p* = 0.38) (Figure S5).

#### 3.2.5 LOS

Five studies involving 375 patients compared the LOS between two groups. There was no significant association between cryotherapy and reduced LOS (MD, −0.36 [95% CI, −1.61 to 0.89]; *p* = 0.57) (Figure S6).

#### 3.2.6 Adverse Event

Thirteen studies involving 569 patients receiving cryotherapy and 565 patients receiving no cryotherapy reported adverse events. Eight studies found no adverse event occurred in either group. There was no significant association between cryotherapy and adverse event (OR, 0.64 [95% CI, 0.31 to 1.32]; *p* = 0.22) (Figure S7).

### 3.3 Continuous Cold Flow Device vs. Cold Pack

#### 3.3.1 Pain

A total of 9 trials compared the VAS between continuous cold flow device and cold pack groups. VAS within POD 3 and over POD 3 were separately investigated in this study. It was found that there was no significant association between cryotherapy devices and VAS either within POD 3 (MD, −0.16 [95% CI, −1.4 to 1.09]; *p* = 0.8) or over POD 3 (MD, −0.40 [95% CI, −1.04 to 0.23]; *p* = 0.21) (Figure S8).

As for the postoperative opioid consumption, two groups showed a comparable result (MD, −5.47 [95% CI, −12.75 to 1.82]; *p* = 0.14) (Figure S9), suggesting the continuous cold flow device did not effectively alleviate postoperative pain when compared to cold pack.

#### 3.3.2 Blood Loss

A total of 3 trials compared the hemoglobin decrease between continuous cold flow device and cold pack groups. The results showed that two cryotherapy modalities had similar hemoglobin decrease (MD, −0.75 [95% CI, −1.793 to 0.42]; *p* = 0.21), demonstrating their comparable effects in reducing blood loss (Figure S10).

#### 3.3.3 ROM

To investigate the association between cryotherapy devices and postoperative ROM after TKA, ROM between continuous cold flow device and cold pack groups were investigated. Continuous cold flow device and cold pack groups had comparable postoperative ROM both within POD 7 (MD, 0.63 [95% CI, −3.91 to 5.17]; *p* = 0.79) and over POD 7 (MD, 3.78 [95% CI, −6.54 to 14.10]; *p* = 0.47) (Figure S11).

#### 3.3.4 Swelling

Six trials involving 420 patients evaluated the knee swelling after TKA. As shown in Figure S12, continuous cold flow device and cold pack groups had similar results regarding the knee swelling (MD, −0.42 [95% CI, −1.06 to 0.22]; *p* = 0.20).

#### 3.3.5 LOS

There was no significant association between cryotherapy devices and LOS (MD, −0.58 [95% CI, −1.45 to 0.29]; *p* = 0.19) (Figure S13).

#### 3.3.6 Adverse Event

Eight studies involving 404 patients in continuous cold flow device group and 397 patients in cold pack group reported adverse events. A total of four cases of adverse events were reported in the continuous cold flow device group, while six case of adverse events occurred in the cold pack group. There was no significant association between cryotherapy devices and adverse event (OR, 0.68 [95% CI, 0.20 to 2.34]; *p* = 0.54) (Figure S14).

### 3.4 Publication Bias

The funnel plots depicting all outcomes are illustrated in Figure S15. No significant asymmetry was observed in the funnel plots, indicating limited evidence for publication bias.

## 4 Discussion

The most important finding of this updated, comprehensive systematic review and meta‐analysis was that cryotherapy can effectively alleviate postoperative pain, reduce blood loss, improve ROM and thus promote the postoperative rehabilitation for TKA patients, but the difference in therapeutic efficacy between the advanced continuous cold flow device and traditional cold pack was not significant. As a result, cryotherapy can be efficient for the rapid rehabilitation of TKA patients, and the traditional cold pack is still recommended for its cost‐efficiency.

### 4.1 Cryotherapy for Rehabilitation After TKA

Prior two systematic reviews investigating the use of cryotherapy in TKA reported that cryotherapy has shown to have some benefits in promoting rehabilitation of patients after TKA [5, 7]. A more recent systematic review published in 2023 by Wyatt et al. included 6 trials concluded that cryotherapy appears to decrease opioid consumption and pain ratings after TKA [42]. However, all above systematic reviews lacked of quantitative analysis, and meta‐analysis with recent RCTs was required to support the conclusion. In this meta‐analysis, we confirmed that cryotherapy can effectively promote patients' rehabilitation after TKA.

With largest number of trials included (18 RCTs), this study's findings have resolved the debate in prior researches regarding the analgesic effects of cryotherapy in TKA. A meta‐analysis by Krampe et al. including 5 RCTs indicated that the cryotherapy demonstrated efficacy in alleviating postoperative pain after TKA [43] while a meta‐analysis by Adie, Naylor, and Harris including 11 RCTs found no benefits in pain reduction by cryotherapy [44]. Another meta‐analysis published by Ni et al. including 7 trials concluded that the cryotherapy reduced pain on the second day after TKA but does not reduce pain on the first day postoperatively [45]. On the contrary, our study found that cryotherapy can decrease the pain score within 3 days after TKA, showing that the analgesic effect of cryotherapy in the worst phase of postoperative pain after TKA (within 3 days after surgery) [46, 47]. The analgesic effect of cryotherapy was further confirmed by the reduced morphine consumption in cryotherapy group when compared to no cryotherapy group. The pain relief effect of cryotherapy may be attributed to the nerve conduction velocity reduction through decreasing the intra‐articular temperature [45, 48].

This study found that cryotherapy can significantly reduce blood loss in TKA patients, as evidenced by decreased hemoglobin decline and drainage. This is the first time that the hemoglobin decline was used as an outcome of measurement in a meta‐analysis to investigate the efficacy of cryotherapy in blood‐saving. The result in decreased drainage was consistent with previous published meta‐analyses, which also reported benefits of cryotherapy in reducing postoperative drainage [44, 45]. The efficacy of cryotherapy in blood‐saving may be attributed to the vasoconstriction stimulated by cryotherapy [8, 45, 49].

### 4.2 Continuous Cold Flow Device vs. Cold Pack

Several cryotherapy devices are available in clinic, including the traditional cryotherapy such as crushed ice in a plastic bag, cold or gel packs, and the advanced cryotherapy devices with continuous cold flow. Continuous cold flow device can provide a steady low temperature with prolonged time and thus theoretically have better clinical efficacy. However, after incorporating all available evidence, we found that the continuous cold flow device and cold pack had similar therapeutic efficacy in patients rehabilitation after TKA. A prior meta‐analysis by Liu et al. including 7 trials also compared continuous cryotherapy and traditional cryotherapy [50]. The results showed that the differences in pain scores, opioid consumption, blood loss, ROM, knee swelling and LOS between continuous cold flow device and cold pack groups were not significant, which was consistent with our results. By including 6 recent high‐quality RCTs, this study further enhances the credibility of this conclusion. We can be speculated that the traditional cold pack can achieve a similar temperature decrease in the knee when compared to the continuous cold flow device. With similar clinical effects, traditional cold pack is still recommended for its cost‐efficiency. However, it is worthy of mention that the effectiveness of cryotherapy is influenced not only by the devices of cryotherapy but also by factors such as the temperature, penetration depth, the timing, and frequency of postoperative cryotherapy application. There have been few trials that monitored the skin temperature or intra‐articular temperature of knee during cryotherapy, making it difficult to directly compare the cooling effects of two cryotherapy modalities.

### 4.3 Cryotherapy‐Related Complications

Concerns about cryotherapy‐related complications still remain. Low temperature or prolonged cooling is theoretically associated with delayed vasodilation and nerve injury, resulting in increased blood loss, severe knee swelling, pain and skin injury [9, 51]. However, cryotherapy demonstrated excellent efficacy in pain relief and hemostasis in this study. Besides, pooled results showed that specific complications related to cryotherapy, such as frostbite injury of skin, delayed healing of wound and wound infection, were comparable between cryotherapy and no cryotherapy groups. The incidence of adverse event was also unrelated to different cryotherapy modalities. It is worth noting that the latest generation of cryotherapy devices incorporates computer‐controlled temperature systems, which may further enhance the safety of cryotherapy [16, 27].

### 4.4 Strengths and Limitations

The strengths of this study are that it includes the largest number of RCTs available in the literature and represents the most comprehensive evaluation of cryotherapy after TKA. This study provides an updated synthesis research of the latest findings and guides clinical practice in the application of cryotherapy after TKA reliably. Limitations exist in this study. First, significant heterogeneity can be found in several outcomes, such as VAS, hemoglobin decrease, drainage and ROM. Clinical heterogeneity among trials, which can be caused by the inconsistent cryotherapy protocols and the difference in patient demographic, may explain the significant heterogeneity. Second, some trials provided inadequate information for secondary outcomes. Thus, it was difficult to perform further subgroup analysis with limited number of studies and sample size. Third, there is notably unclear risk of bias in some included studies due to poorly described methodology. The quality of included trials may influence the conclusion of this study. Fourth, more persuasive studies, such as multicenter RCTs and real‐world studies, can be beneficial to resolve the controversies in cryotherapy.

## 5 Conclusion

In this updated meta‐analysis of RCTs, the use of cryotherapy can effectively alleviate postoperative pain, reduce blood loss, improve ROM and thus promote the postoperative rehabilitation for TKA patients. There was no evidence to support that the continuous cold flow device had better efficacy in promoting the patients' recovery than cold packs. These findings support the use of cryotherapy for the rapid rehabilitation of TKA patients, and the traditional cold pack is still recommended for its cost‐efficiency.

## Author Contributions

All authors listed above meet the authorship criteria according to the latest guidelines of the International Committee of Medical Journal Editors and are in agreement with the manuscript.

## Conflicts of Interest

The authors declare no conflicts of interest.

## Supporting information


**Figure S1.** Forest plot for opioid consumption (cryotherapy vs. no cryotherapy).

**Figure S2.** Forest plot for hemoglobin decrease (cryotherapy vs. no cryotherapy).

**Figure S3.** Forest plot for drainage (cryotherapy vs. no cryotherapy).

**Figure S4.** Forest plot for ROM (cryotherapy vs. no cryotherapy).

**Figure S5.** Forest plot for swelling (cryotherapy vs. no cryotherapy).

**Figure S6.** Forest plot for LOS (cryotherapy vs. no cryotherapy).

**Figure S7.** Forest plot for adverse event (cryotherapy vs. no cryotherapy).

**Figure S8.** Forest plot for pain score (continuous cold flow device vs. cold pack).

**Figure S9.** Forest plot for opioid consumption (continuous cold flow device vs. cold pack).

**Figure S10.** Forest plot for hemoglobin decrease (continuous cold flow device vs. cold pack).

**Figure S11.** Forest plot for ROM (continuous cold flow device vs. cold pack).

**Figure S12.** Forest plot for swelling (continuous cold flow device vs. cold pack).

**Figure S13.** Forest plot for LOS (continuous cold flow device vs. cold pack).

**Figure S14.** Forest plot for adverse event (continuous cold flow device vs. cold pack).

**Figure S15.** Funnel plots. (A) Cryotherapy vs. no cryotherapy on pain score. (B) Cryotherapy vs. no cryotherapy on pain score‐ Subgroup analysis. (C) Cryotherapy vs. no cryotherapy on swelling. (D) Cryotherapy vs. no cryotherapy on ROM. (E) Cryotherapy vs. no cryotherapy on opioid consumption. (F) Cryotherapy vs. no cryotherapy on drainage. (G) Cryotherapy vs. no cryotherapy on hemoglobin decrease. (H) Cryotherapy vs. no cryotherapy on LOS. (I) Cryotherapy vs. no cryotherapy on adverse event. (J) Continuous cold flow device vs. cold pack on pain score. (K) Continuous cold flow device vs. cold pack on LOS. (L) Continuous cold flow device vs. cold pack on ROM. (M) Continuous cold flow device vs. cold pack on opioid consumption. (N) Continuous cold flow device vs. cold pack on swelling. (O) Continuous cold flow device vs. cold pack on adverse event. (P) Continuous cold flow device vs. cold pack on hemoglobin decrease.

## Data Availability

The authors have nothing to report.
