# Supplementary figures and images for: Cryotherapy for Rehabilitation After Total Knee Arthroplasty: A Comprehensive Systematic Review and Meta‐Analysis
Source: Orthop Surg. 2024 Oct 14;16(12):2897–915. doi: 10.1111/os.14266 (PMC11608804; doi:10.1111/os.14266)

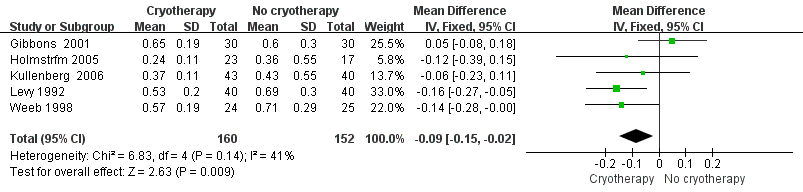

Supplement: Supplementary file 1 — Figure S1. Forest plot for opioid consumption (cryotherapy vs. no cryotherapy). [file OS-16-2897-s004.png]

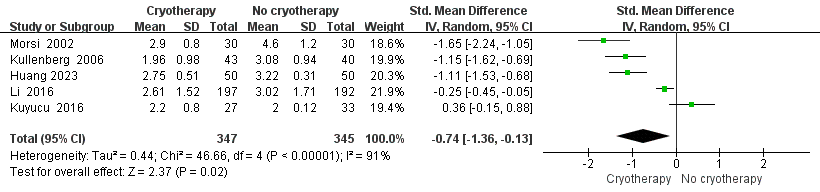

Supplement: Supplementary file 2 — Figure S2. Forest plot for hemoglobin decrease (cryotherapy vs. no cryotherapy). [file OS-16-2897-s013.png]

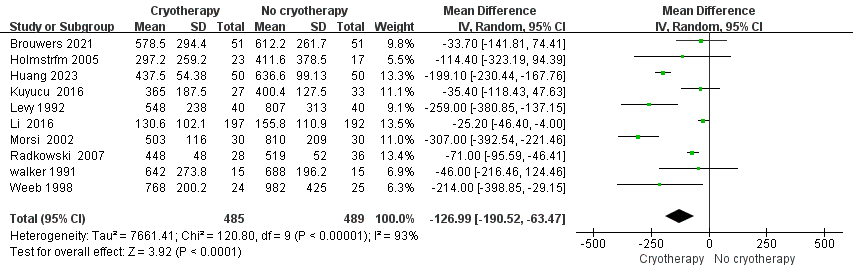

Supplement: Supplementary file 3 — Figure S3. Forest plot for drainage (cryotherapy vs. no cryotherapy). [file OS-16-2897-s014.png]

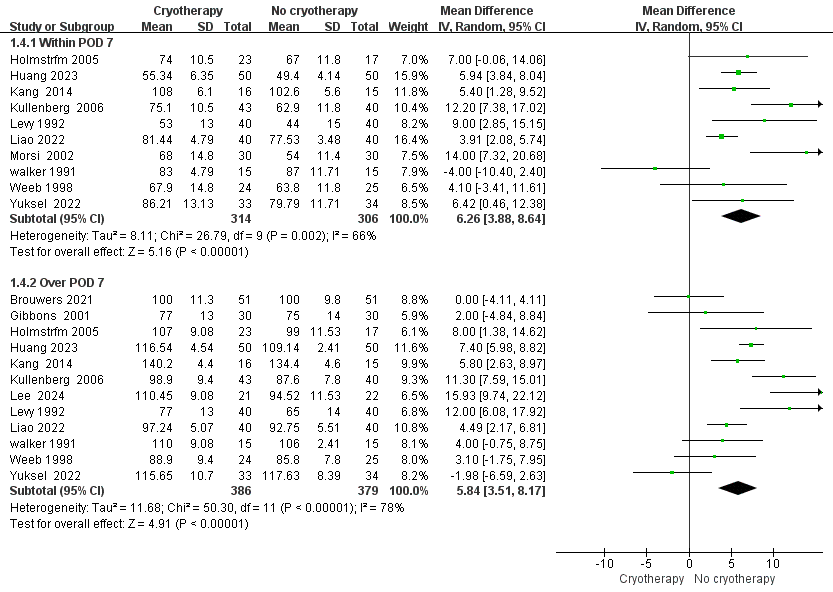

Supplement: Supplementary file 4 — Figure S4. Forest plot for ROM (cryotherapy vs. no cryotherapy). [file OS-16-2897-s008.png]

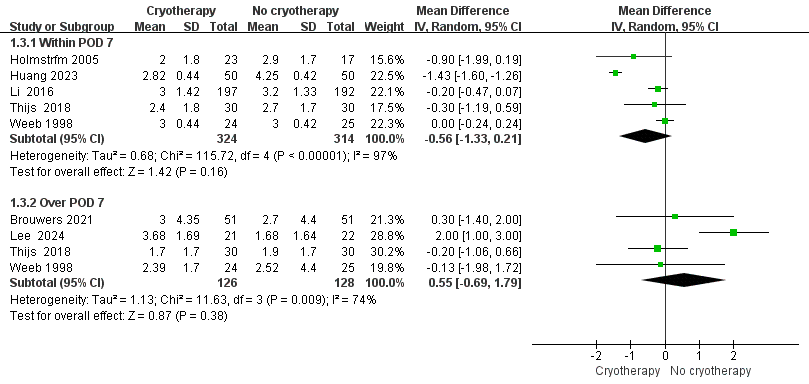

Supplement: Supplementary file 5 — Figure S5. Forest plot for swelling (cryotherapy vs. no cryotherapy). [file OS-16-2897-s007.png]

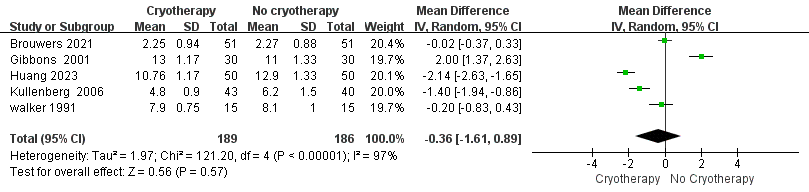

Supplement: Supplementary file 6 — Figure S6. Forest plot for LOS (cryotherapy vs. no cryotherapy). [file OS-16-2897-s003.png]

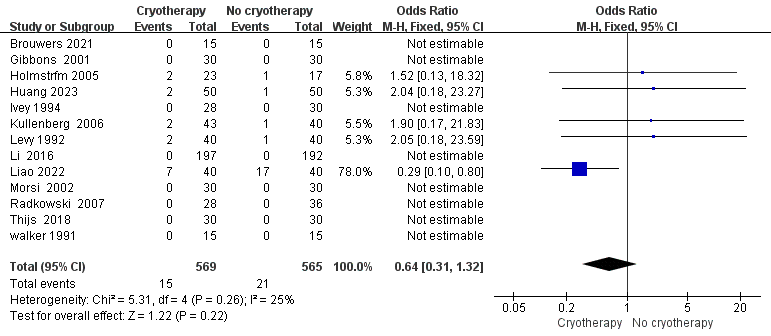

Supplement: Supplementary file 7 — Figure S7. Forest plot for adverse event (cryotherapy vs. no cryotherapy). [file OS-16-2897-s006.png]

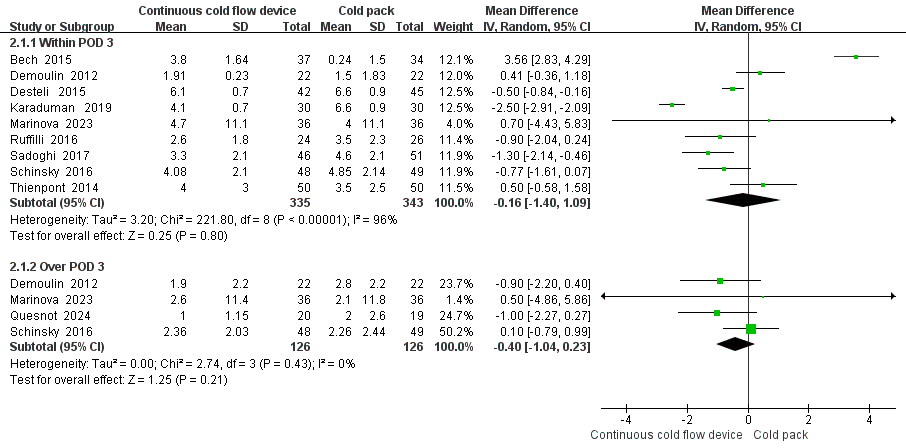

Supplement: Supplementary file 8 — Figure S8. Forest plot for pain score (continuous cold flow device vs. cold pack). [file OS-16-2897-s011.png]

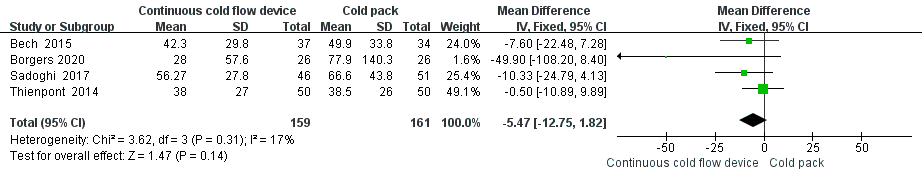

Supplement: Supplementary file 9 — Figure S9. Forest plot for opioid consumption (continuous cold flow device vs. cold pack). [file OS-16-2897-s005.png]

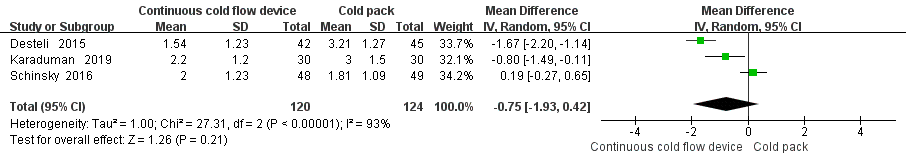

Supplement: Supplementary file 10 — Figure S10. Forest plot for hemoglobin decrease (continuous cold flow device vs. cold pack). [file OS-16-2897-s010.png]

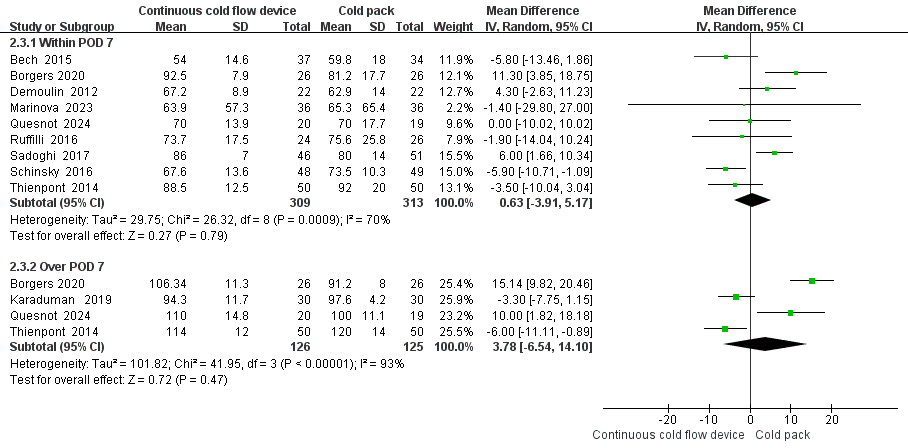

Supplement: Supplementary file 11 — Figure S11. Forest plot for ROM (continuous cold flow device vs. cold pack). [file OS-16-2897-s012.png]

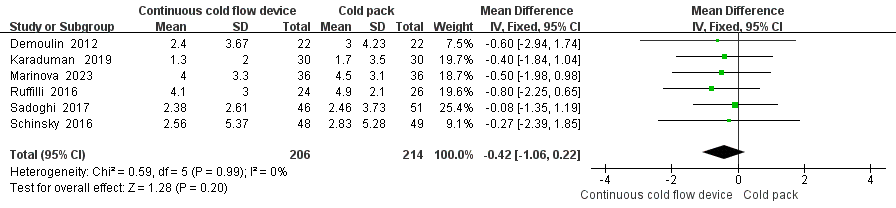

Supplement: Supplementary file 12 — Figure S12. Forest plot for swelling (continuous cold flow device vs. cold pack). [file OS-16-2897-s015.png]

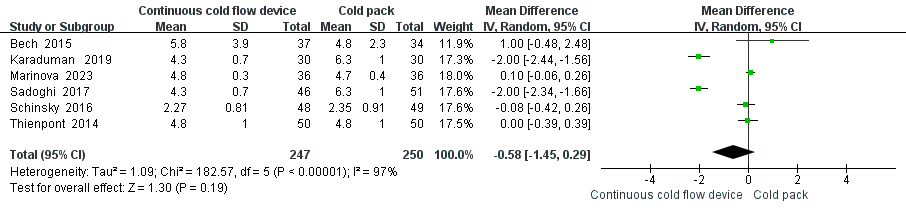

Supplement: Supplementary file 13 — Figure S13. Forest plot for LOS (continuous cold flow device vs. cold pack). [file OS-16-2897-s002.png]

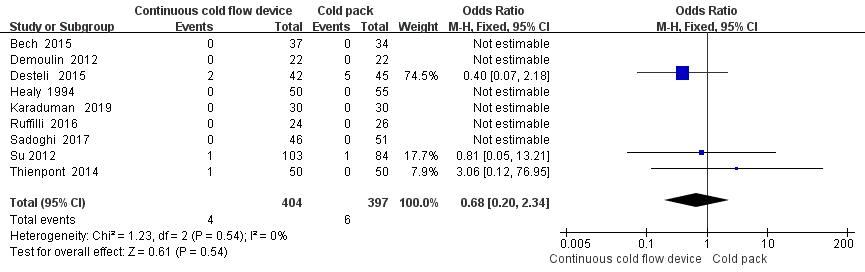

Supplement: Supplementary file 14 — Figure S14. Forest plot for adverse event (continuous cold flow device vs. cold pack). [file OS-16-2897-s001.png]

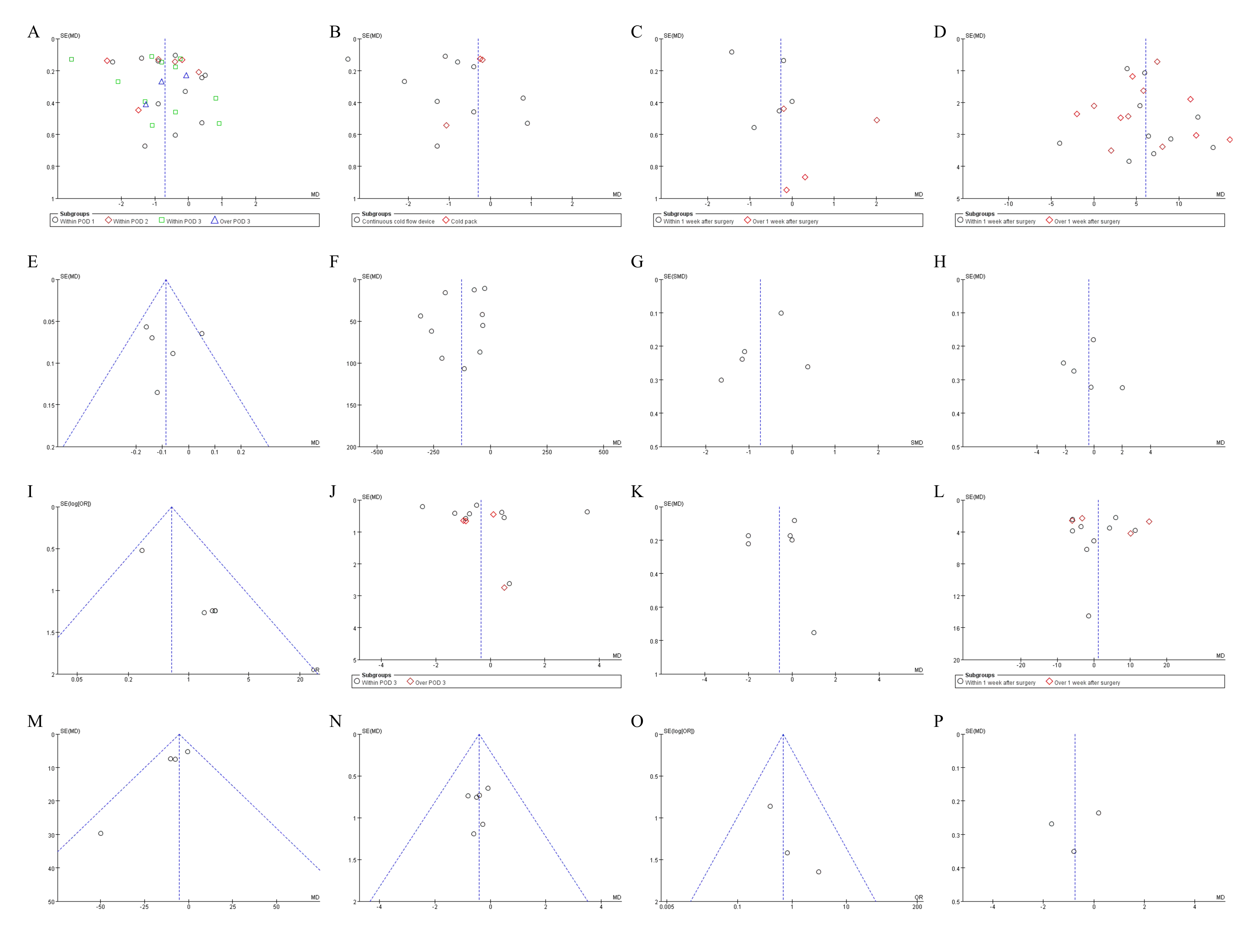

Supplement: Supplementary file 15 — Figure S15. Funnel plots. (A) Cryotherapy vs. no cryotherapy on pain score. (B) Cryotherapy vs. no cryotherapy on pain score‐ Subgroup analysis. (C) Cryotherapy vs. no cryotherapy on swelling. (D) Cryotherapy vs. no cryotherapy on ROM. (E) Cryotherapy vs. no cryotherapy on opioid consumption. (F) Cryotherapy vs. no cryotherapy on drainage. (G) Cryotherapy vs. no cryotherapy on hemoglobin decrease. (H) Cryotherapy vs. no cryotherapy on LOS. (I) Cryotherapy vs. no cryotherapy on adverse event. (J) Continuous cold flow device vs. cold pack on pain score. (K) Continuous cold flow device vs. cold pack on LOS. (L) Continuous cold flow device vs. cold pack on ROM. (M) Continuous cold flow device vs. cold pack on opioid consumption. (N) Continuous cold flow device vs. cold pack on swelling. (O) Continuous cold flow device vs. cold pack on adverse event. (P) Continuous cold flow device vs. cold pack on hemoglobin decrease. [file OS-16-2897-s009.png]
